# RE‐START: Exploring the effectiveness of anti‐calcitonin gene‐related peptide resumption after discontinuation in migraine

**DOI:** 10.1111/ene.16203

**Published:** 2024-01-25

**Authors:** Celia Romero del Rincón, Alicia Gonzalez‐Martinez, Sonia Quintas, David García‐Azorín, Iris Fernández Lázaro, Angel Luis Guerrero‐Peral, Yesica Gonzalez Osorio, Sonia Santos‐Lasaosa, Carmen González Oria, Norberto Sánchez Rodríguez, Fernando Iglesias Díez, Ana Echavarría Íñiguez, Sendoa Gil Luque, Mariano Huerta‐Villanueva, Sergio Campoy Díaz, Albert Muñoz‐Vendrell, Alberto Lozano Ros, Antonio Sánchez‐Soblechero, Fernando Velasco Juanes, Izaro Kortazar‐Zubizarreta, Amaya Echeverría, Jaime Rodríguez‐Vico, Alex Jaimes Sánchez, Andrea Gómez García, Noemí Morollón Sánchez‐Mateos, Robert Belvis, María Pilar Navarro Pérez, Juan Carlos García‐Moncó, María Rocío Álvarez Escudero, Nuria Montes, Ana Beatriz Gago‐Veiga

**Affiliations:** ^1^ Department of Neurology, Hospital Universitario de La Princesa & Instituto de Investigación Sanitaria Princesa (IIS‐Princesa) Universidad Autónoma de Madrid (UAM) Madrid Spain; ^2^ Headache Unit Hospital Clínico Universitario de Valladolid Valladolid Spain; ^3^ Department of Medicine Universidad de Valladolid Valladolid Spain; ^4^ Headache Unit Hospital Clínico Universitario Lozano Blesa Zaragoza Spain; ^5^ Headache Unit Hospital Virgen del Rocío Sevilla Spain; ^6^ Headache Unit Hospital Clínico Universitario de Burgos Burgos Spain; ^7^ Neurology Department of Neurology Hospital de Viladecans‐IDIBELL Viladecans, Barcelona Spain; ^8^ Servicio de Neurología, Unidad de Cefaleas, Hospital Universitari de Bellvitge‐IDIBELL L'Hospitalet de Llobregat Barcelona Spain; ^9^ Headache Unit Hospital Universitario Gregorio Marañón Madrid Spain; ^10^ Headache Unit Hospital Universitario de Cruces Barakaldo, Vizcaya Spain; ^11^ Department of Neurology, Hospital de Álava, Bioaraba Health Research Institute Araba University Hospital‐Txagorritxu Vitoria‐Gasteiz Spain; ^12^ Headache Unit Hospital Fundación Jiménez Díaz Madrid Spain; ^13^ Hospital de la Santa Creu i Sant Pau Barcelona Spain; ^14^ Hospital Obispo Polanco de Teruel Instituto Investigación Sanitaria Aragón Zaragoza Spain; ^15^ Hospital Universitario de Basurto Bilbao Spain; ^16^ Hospital Universitario Central de Asturias Oviedo Spain; ^17^ Unidad de Metodología Instituto de Investigación Sanitaria La Princesa (IIS‐IP) Madrid Spain; ^18^ Servicio de Reumatología Hospital Universitario La Princesa Madrid Spain; ^19^ Plant Physiology, Pharmaceutical and Health Sciences Department, Faculty of Pharmacy Universidad San Pablo‐CEU, CEU‐Universities Boadilla del Monte Spain

**Keywords:** CGRP, effectiveness, migraine, response, resumption

## Abstract

**Background and purpose:**

According to the latest European guidelines, discontinuation of monoclonal antibodies against calcitonin gene‐related peptide (anti‐CGRP MAb) may be considered after 12–18 months of treatment. However, some patients may worsen after discontinuation. In this study, we assessed the response following treatment resumption.

**Methods:**

This was a prospective study conducted in 14 Headache Units in Spain. We included patients with response to anti‐CGRP MAb with clinical worsening after withdrawal and resumption of treatment. Numbers of monthly migraine days (MMD) and monthly headache days (MHD) were obtained at four time points: before starting anti‐CGRP MAb (T‐baseline); last month of first treatment period (T‐suspension); month of restart due to worsening (T‐worsening); and 3 months after resumption (T‐reintroduction). The response rate to resumption was calculated. Possible differences among periods were analysed according to MMD and MHD.

**Results:**

A total of 360 patients, 82% women, with a median (interquartile range [IQR]) age at migraine onset of 18 (12) years. The median (IQR) MHD at T‐baseline was 20 (13) and MMD was 5 (6); at T‐suspension, the median (IQR) MHD was 5 (6) and MMD was 4 (5); at T‐worsening, the median (IQR) MHD was 16 (13) and MMD was 12 (6); and at T‐reintroduction, the median (IQR) MHD was 8 (8) and MHD was 5 (5). In the second period of treatment, a 50% response rate was achieved by 57.4% of patients in MHD and 65.8% in MMD. Multivariate models showed significant differences in MHD between the third month after reintroduction and last month before suspension of first treatment period (*p* < 0.001).

**Conclusion:**

The results suggest that anti‐CGRP MAb therapy is effective after reintroduction. However, 3 months after resumption, one third of the sample reached the same improvement as after the first treatment period.

## INTRODUCTION

Migraine is a chronic neurological condition distinguished by repetitive, highly incapacitating and unpredictable headache attacks combined with various other neurological symptoms, affecting about one billion people worldwide per year [1], with an estimated global prevalence of 14% on the basis of the 2016 Global Burden of Disease study [2, 3].

Over the past decade, the landscape of migraine treatment has undergone a significant transformation with the introduction of monoclonal antibodies (MAb) targeting calcitonin gene‐related peptide (CGRP) as a prophylactic approach [4, 5, 6, 7, 8]. Currently, four anti‐CGRP MAb (galcanezumab, erenumab, fremanezumab and eptinezumab) are available. Despite the increasing evidence of the real‐world effectiveness and safety of these drugs, the optimal treatment duration is unknown. Moreover, to what extent discontinuation after a successful therapy period may impact the effectiveness of anti‐CGRP MAb reintroduction remains unclear [4, 9, 10].

According to the European Headache Federation (EHF) guidelines, published in 2019, prophylactic migraine treatment should last 6–12 months when it is effective, with a discontinuation attempt afterwards [11]. However, the arrival of anti‐CGRP MAb in the migraine management landscape prompted a re‐evaluation of this recommendation, as real‐world studies highlighted that a substantial proportion of patients experienced a worsening in their migraine courses after treatment discontinuation [12, 13, 14]. In light of this, the EHF revised its guidelines in 2022, and the current expert consensus includes the following: (i) a pause in treatment after 12–18 months, and resumption in case of worsening after discontinuation, and (ii) if deemed necessary, continuation of treatment as long as needed [15].

Previous studies, with limited sample sizes, suggest that the improvement after treatment resumption does not differ from the response in the initial treatment period [16]. Nevertheless, it is important to acknowledge that some patients might show a milder response in comparison to first treatment period [12, 13, 16, 17]. To date, it remains unclear whether treatment resumption can achieve a clinical response comparable to that of the first period. In this study, we aimed to evaluate the second treatment period response and to compare it to the initial response to anti‐CGRP MAb treatment.

## METHODS

### Study design and participants

This was an observational prospective analytical multicentric study. The study was conducted in 14 Spanish Headache Units. The study period ranged between January 2020 and May 2022.

### Eligibility criteria

Patients were included if: (i) they were aged over 18 years; (ii) they had a diagnosis of high‐frequency episodic migraine (HFEM) or chronic migraine (CM) according to the International Classification of Headache Disorders 3rd edition (ICHD‐3) criteria [18]; (iii) they were treated with anti‐CGRP MAb treatment after fulfilling the national prescription criteria for this, which include eight or more MMDs and lack of response to at least three preventive medications, each taken at sufficient doses for a minimum of 3 months, one of these medications being onabotulinumtoxinA in the case of CM [10, 19]. Patients could have received galcanezumab, erenumab or fremanezumab; eptinezumab was not yet commercialized during this study); (iv) they were receiving anti‐CGRP MAb treatment for at least 12 months and had good clinical response according to the responsible physician; and (v) they had migraine worsening after discontinuation that led to restarting the anti‐CGRP MAb. The decision to restart depended on each clinician's criteria based on their clinical practice, with different rates of and time to worsening, as a homogenous criterion for reintroduction is not defined in the different guidelines.

Patients were excluded if they had another primary or secondary headache disorder, except for low‐frequency tension‐type headache or medication overuse headache.

### Study variables

Demographic and baseline information was collected, including age, sex, age at migraine onset, migraine type (high‐frequency episodic or chronic), age at anti‐CGRP MAb initiation, presence of aura, oppressive periocular pain, headache topography, medication overuse with nonsteroidal anti‐inflammatory drugs (NSAIDs), opioids or triptans, number of prior preventive drugs attempted and type of anti‐CGRP MAb started. Numbers of monthly headache days (MHD) and monthly migraine days (MMD), and pain intensity at each of the four study time points were collected as study variables. A migraine day was defined as a day with moderate or severe pain that lasts at least 4 h and had to meet migraine features defined by the ICHD‐3, such as photophobia, phonophobia, nausea, vomiting, or worsening with physical activity, or a day with a headache that is successfully treated by an acute headache medication. Headache day was defined as a day with pain that did not meet the previous criteria. Patients had been trained before starting the treatment. Pain intensity information was collected with a rating from 0 to 100 to assess differences more accurately.

### Study procedures

**FIGURE 1:**
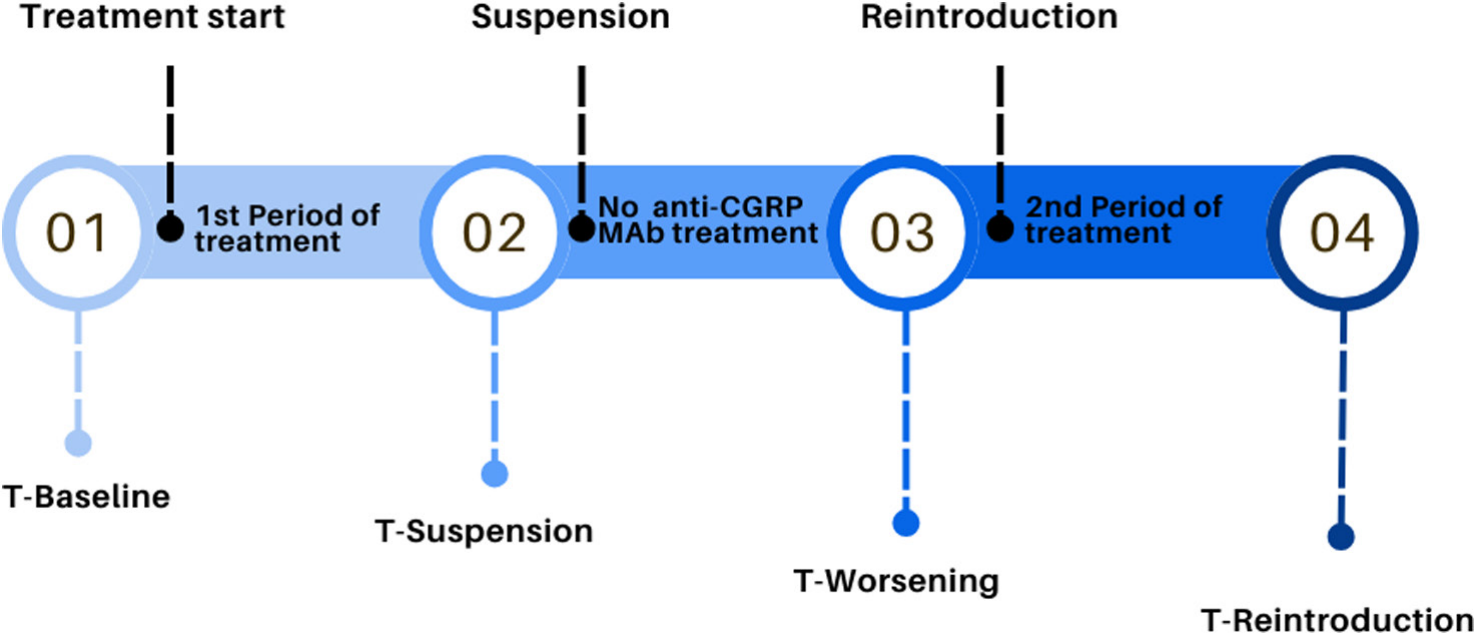
Flowchart describing the four time points of our study: (1) month before initiating monoclonal antibodies against calcitonin gene‐related peptide (MAb) treatment (T‐Baseline); (2) final month of the first period (T‐Suspension); (3) once discontinued, during worsening in the last month before deciding to resume treatment (T‐Worsening); and (4) the third month after treatment reintroduction (T‐Reintroduction).

Patients were evaluated in terms of MMD and MHD. Data were collected at four time points (Figure 1). Headache diaries were prospectively collected as part of clinical practice in specialized Headache Units.

### Outcome measures

The primary objective of this study was to assess the effectiveness of resumption of anti‐CGRP MAb regarding the number of patients achieving 30%, 50% and 75% response rates considering both MMD and MHD.

Secondary objectives included the comparison of effectiveness after the first and second treatment period regarding MMDs and MHDs. This endpoint was calculated as the difference between T‐Reintroduction (third month after treatment reintroduction) and T‐Suspension (final month of the first period) in MMDs and MHDs. Positive response was considered if the result of this subtraction was ≤0. Patients with an increase in the number of MMD or MHD during the second period when compared to the first were divided into three subgroups (1–4 days: mild; 5–10 days: moderate; >10 days: severe).

As a third exploratory objective, we aimed to identify the factors that influenced the change in headache and migraine days between the end of the first period and the 3 months after reintroduction (second period).

### Statistical analysis

Demographic and baseline characteristics were described using medians with interquartile ranges (IQRs) for continuous variables, and percentages for categorical data. MHD and MMD were not normally distributed, and variances were heterogeneous according to Kolmogorov–Smirnov and Levene's tests, respectively. These variables were fitted to a Gamma distribution according to Akaike's Information Criterion (R package: risk Distributions) [20]. The responder rate was calculated as a percent reduction from baseline in the number of MHD and MMD in each treatment period (30%, 50% and 75% reduction from baseline): first treatment period T‐Suspension to T‐Baseline (month before initiating anti‐CGRP MAb in the first period) and second treatment period T‐Reintroduction to T‐Worsening (final month of the first period). The temporal analyses of MHD and MMD by GzLMMs (generalized linear mixed models) were performed using R‐libraries glmmTMB [21] considering temporal autocorrelation with AR1 matrix covariance. Pearson's chi‐squared and Fisher's exact tests were employed to examine associations between categorical variables. The Kruskal–Wallis rank sum test was used to compare non‐parametric continuous variables among multiple groups. To comprehensively understand the combined impacts of the variables under investigation, two separate multivariate models were constructed. The first model aimed to determine the independent variables associated with the dependent variable MHD, while the second model focused on MMD at the four time points. The reference time point in the analysis was T‐Suspension. Statistical analyses were conducted using R v.4.2.2.

A formal sample size calculation was not performed; instead, it was approximated based on the data available in the literature at the time of the study onset.

### Ethics

This study was approved by the Ethics Committee of the University Clinical Hospital of Valladolid (PI‐GR‐22‐2838) and is part of ClinicalTrials.gov identifier: NCT05232942. The study was conducted in accordance with the ethical principles of the Declaration of Helsinki.

## RESULTS

A total of 360 patients were included in the study, 82% of whom (293/360) were women, with a median (IQR) age at migraine onset of 18 (12.07) years and a median (IQR) age at the initiation of MAb of 48.8 (13.64) years. Regarding migraine type, 73% (262/360) met the criteria for CM, whereas the remaining patients met the criteria for HFEM. Twenty per cent of patients (70/358) had migraine with aura. Anti‐CGRP MAb therapy was initiated with erenumab in 42.8% (154/360), fremanezumab in 31.7% (114/360) and galcanezumab in 25.6% of patients (92/360).

Concerning migraine characteristics, 72% (136/190) presented with hemicranial pain, and 9.2% (33/360) had oppressive periocular pain. We observed a median (IQR) of 5 (2) previously failed preventive treatments tested. Medication overuse headache criteria were fulfilled by 45% of patients (163/360), the overused drug being NSAIDs in 34% (124/360), opioids in 1.1% (4/360) and triptans in 41% (146/360).

**FIGURE 2:**
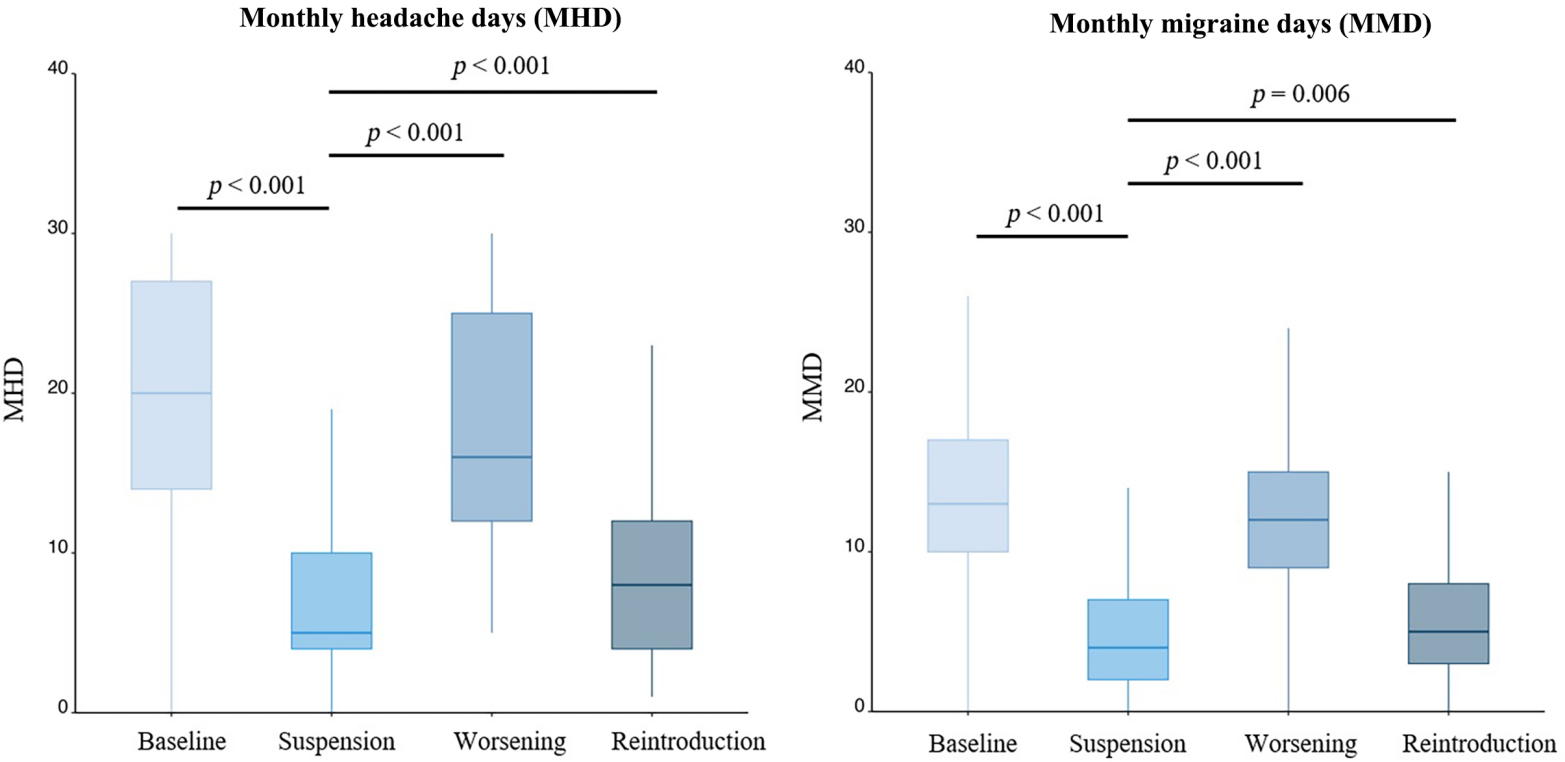
Box plot illustrating the evolution of monthly migraine days (MMD) and monthly headache days (MHD) at the four time points of the study. T‐suspension (final month of the first period) was established as a reference for statistical analysis. The data reveal a pattern in which MMD decrease following the initial introduction of treatment, increase after suspension, and subsequently decrease again upon reintroduction.

In the evaluation of the changes in migraine frequency throughout the treatment evolution, we observed the following median (IQR) values: 20 (13) MHD and 13 (7) MMD in the month before initiating MAb treatment (T‐Baseline). Subsequently, a median (IQR) of 5 (6) MHD and 4 (5) MMD were recorded in the month prior to suspension of treatment (T‐Suspension). During the phase of worsening, in the month before antibody reintroduction, patients exhibited 16 (13) MHD and 12 (6) MMD (T‐Worsening). Three months after the reintroduction of the antibody treatment, patients demonstrated improvement, with a median (IQR) of 8 (8) MHD and 5 (5) MMD (T‐Reintroduction) (Figure 2). In terms of pain intensity, a similar trend was observed: 80 (14) at T‐Baseline, 60 (20) at T‐Suspension, 73 (15) at T‐Worsening and 65 (25) at T‐Reintroduction. The median (IQR) time between discontinuation of the first period and reintroduction of treatment was 4.4 (3.5) months.

**FIGURE 3:**
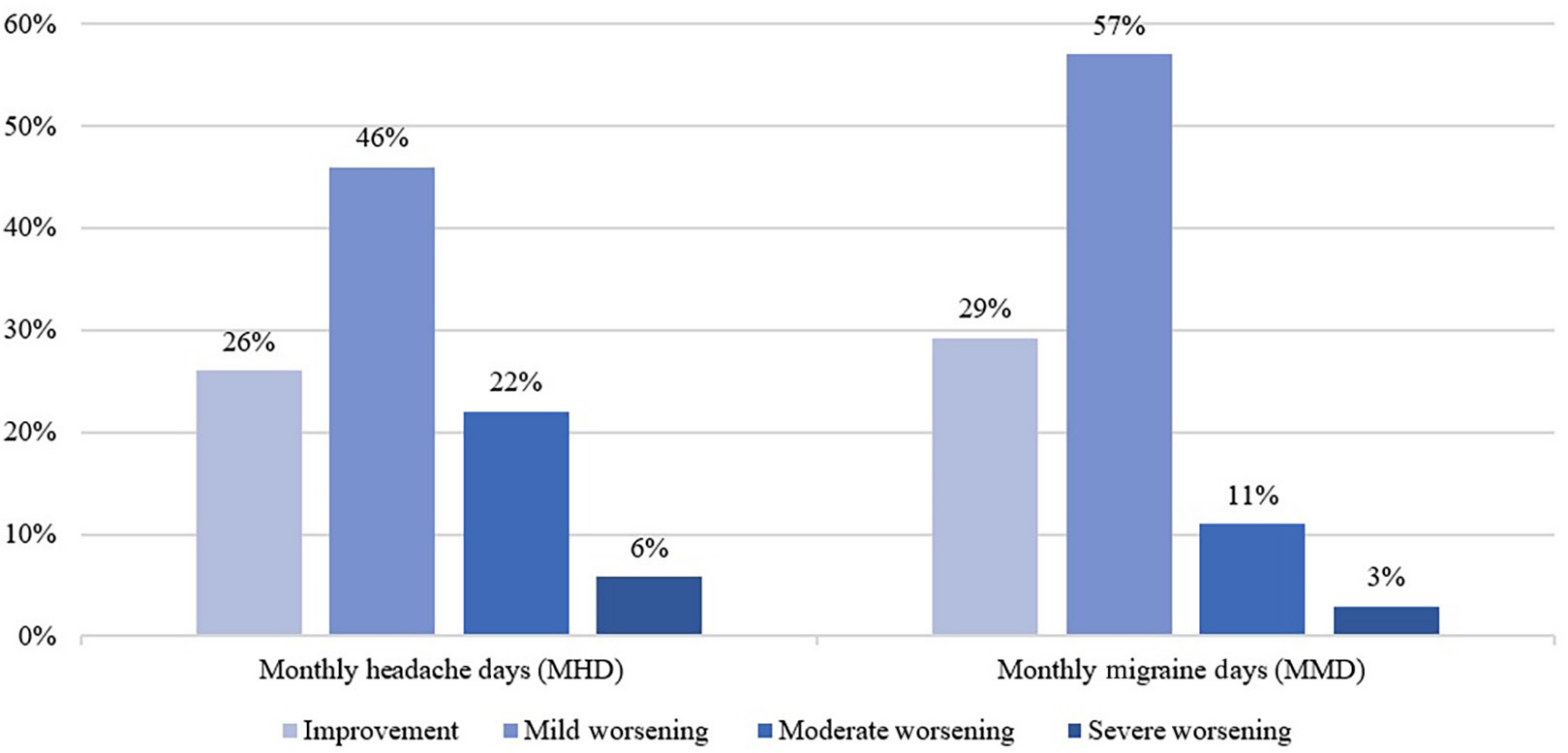
Graphical representation of patient response to treatment reintroduction (with respect to the end of the first period). On the left, monthly headache days (MHD) are shown on the left, and monthly migraine days (MMD) on the right. Each section includes four bars, each reflecting patients achieving improvement or worsening (in three subcategories) compared with the end of the first treatment period.

The proportion of patients who achieved a 30% reduction at the end of the first period (T‐Suspension) in MHD was 89.4% (322/358), while in MMD it was 84.4% (304/342). All the patients presented a clinically meaningful improvement that led the responsible physician to maintain the drug for the entire treatment period. The response rate to the second treatment period was calculated. Regarding MHD, 75.3% (271/358) of the patients had a response rate of 30%, 54.7% (197/358) a response rate of 50%, and 18.1% (65/358) a response rate of 75%. Considering MMD, 80.8% (291/347) of the patients had a response rate of 30%, 65.8% (237/347) a response rate of 50%, and 21.4% (77/347) a response rate of 75%. The graphical representation of patient response to treatment reintroduction is included in Figure 3.

Baseline demographic and headache characteristics influencing change in MHD and MMD were analysed. T‐Baseline was taken as the reference to analyse MMD and MHD at the remaining time points.

Concerning change in MHD, patients with CM experienced a greater number of MHD (*p* < 10^−4^, β‐coefficient = 0.34). The presence of oppressive periocular pain was associated with increased number of MHD (*p* < 10^−3^, β‐coefficient = 0.22). A higher number of prior treatments correlated with more headache days (*p* = 0.034, β‐coefficient = 0.01). Furthermore, patients with medication overuse exhibited a greater number of MHD with NSAIDs (*p* < 10^−4^, β‐coefficient = 0.02), opioids (*p* = 0.03, β‐coefficient = 0.01) and triptans (*p* < 10^−4^, β‐coefficient = 0.020) when they met the criteria. Additionally, other variables such as sex, age of migraine onset, age at the initiation of anti‐CGRP MAb treatment, topography and presence of aura were also examined, but no significant associations were observed.

Regarding change in MMD, we observed that this was influenced by certain factors. Age at migraine onset (*p* = 0.05, *β*‐coeff = 0.004) has been correlated with an increase in number of MMD, with patients with an earlier onset of migraine having had a greater number of migraine days per month. Additionally, migraine type has also shown a significant effect on MMD (*p* = 0.002, β‐coefficient = 0.138), with patients with CM having a higher number of migraine days per month compared to other migraine types. Furthermore, we have observed that patients with medication overuse, specifically NSAIDs (*p* < 10^−4^, β‐coefficient = 0.02), opioids (*p* = 0.01, β‐coefficient = 0.017) and triptans (*p* < 10^−4^, β‐coefficient = 0.03) also experience an increase in number of MMD. However, the other variables studied have not shown significant associations with MMD.

To comprehensively understand the combined impacts of the variables under investigation, two separate multivariate models were constructed. The first model aimed to determine the independent variables associated with the dependent variable MHD (Table 1), while the second model focused on MMD (Table 2) at the four time points. The outcomes reveal a significant difference in MHD between T‐Reintroduction and T‐Suspension (*p* < 0.001, β‐coefficient = 0.67), with a noticeable trend in MMD although not statistically significant (*p* = 0.067, β‐coefficient = 0.07).

**TABLE 1:** Multivariate model for monthly headache days at the four time points and predictive variables for these.

Variables	*β*‐coefficient	95% CI	*p* value
T‐Worsening	0.67	0.60, 0.74	<0.001
T‐Reintroduction	0.16	0.09, 0.23	<0.001
T‐Baseline	0.67	0.59, 0.75	<0.001
Migraine type: CM	0.19	0.10, 0.28	<0.001
Oppressive periocular pain	0.13	0.02, 0.24	0.019
Number of prior preventive treatments	0	−0.01, 0.02	0.947
MOH: NSAIDs	0.03	0.02, 0.03	<0.001
MOH: opioids	0	−0.01, 0.02	0.663
MOH: triptans	0.02	0.02, 0.03	<0.001

Abbreviations: CI, confidence interval; CM, chronic migraine; MOH, medication overuse headache; NSAID, nonsteroidal anti‐inflammatory drugs; T‐Baseline, month before initiating anti‐CGRP MAb treatment; T‐Worsening, last month before the resumtion of treatment; T‐Reintroduction, the third month after anti‐CGRP MAb resumption.

**TABLE 2:** Multivariate model for monthly migraine days at the four time points and predictive variables for these.

Variables	*β*‐coefficient	95% CI	*p* value
T‐Worsening	0.61	0.53, 0.69	<0.001
T‐Reintroduction	0.07	−0.01, 0.14	0.069
T‐Baseline	0.6	0.52, 0.69	<0.001
Migraine type: CM	0.09	0.00, 0.18	0.048
NSAIDs overuse	0.02	0.01, 0.02	<0.001
Opioids overuse	0	−0.01, 0.02	0.669
Triptans overuse	0.04	0.03, 0.04	<0.001

Abbreviations: CI, confidence interval; CM, chronic migraine; NSAIDs, nonsteroidal anti‐inflammatory drugs; T‐Baseline, month before initiating anti‐CGRP MAb treatment; T‐Worsening, last month before the resumption of treatment; T‐Reintroduction, the third month after anti‐CGRP MAb reintroduction.

**FIGURE 4:**
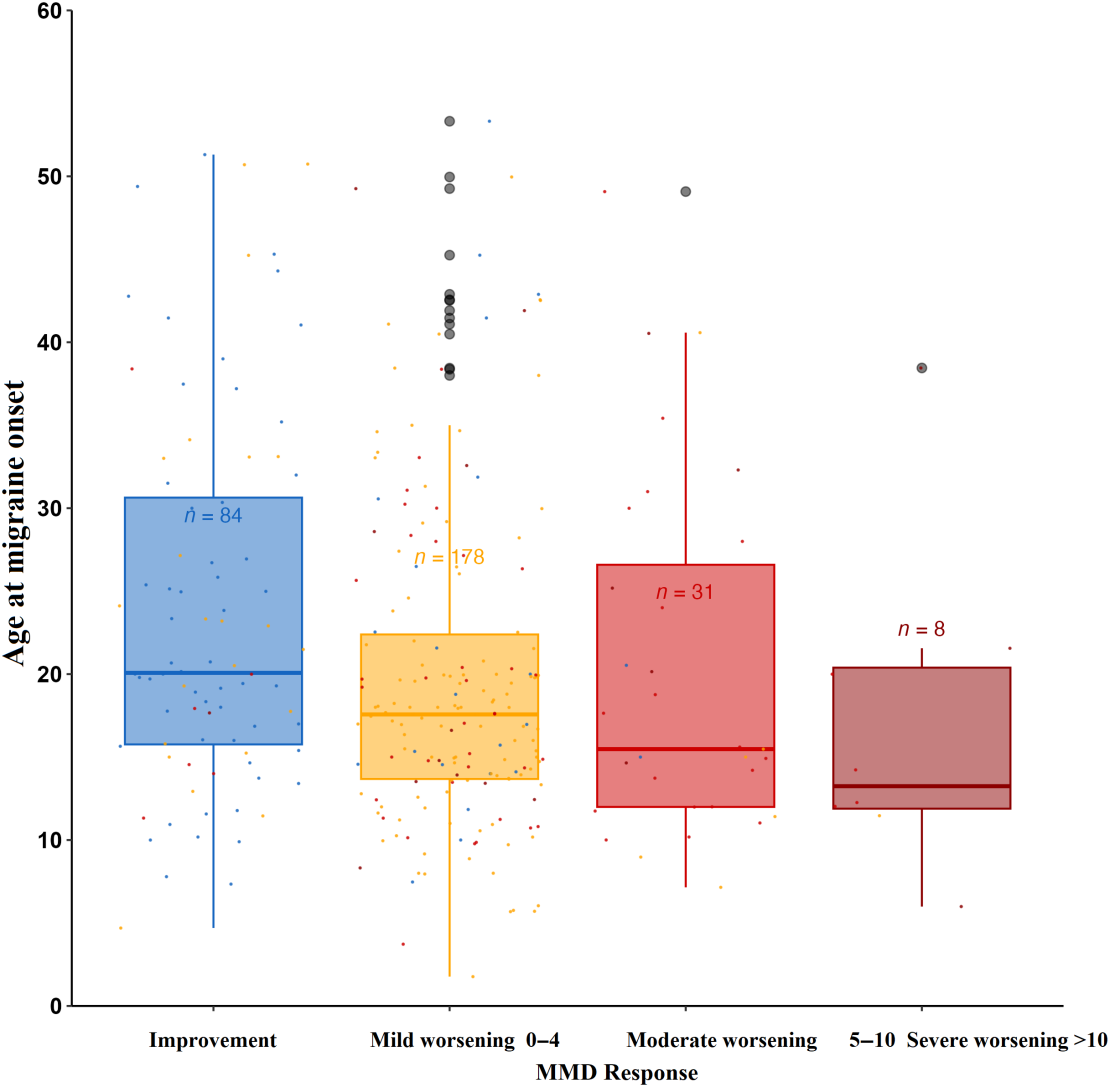
Relationship between age of onset and differences between the two period treatments. MMD, monthly migraine days.

**FIGURE 5:**
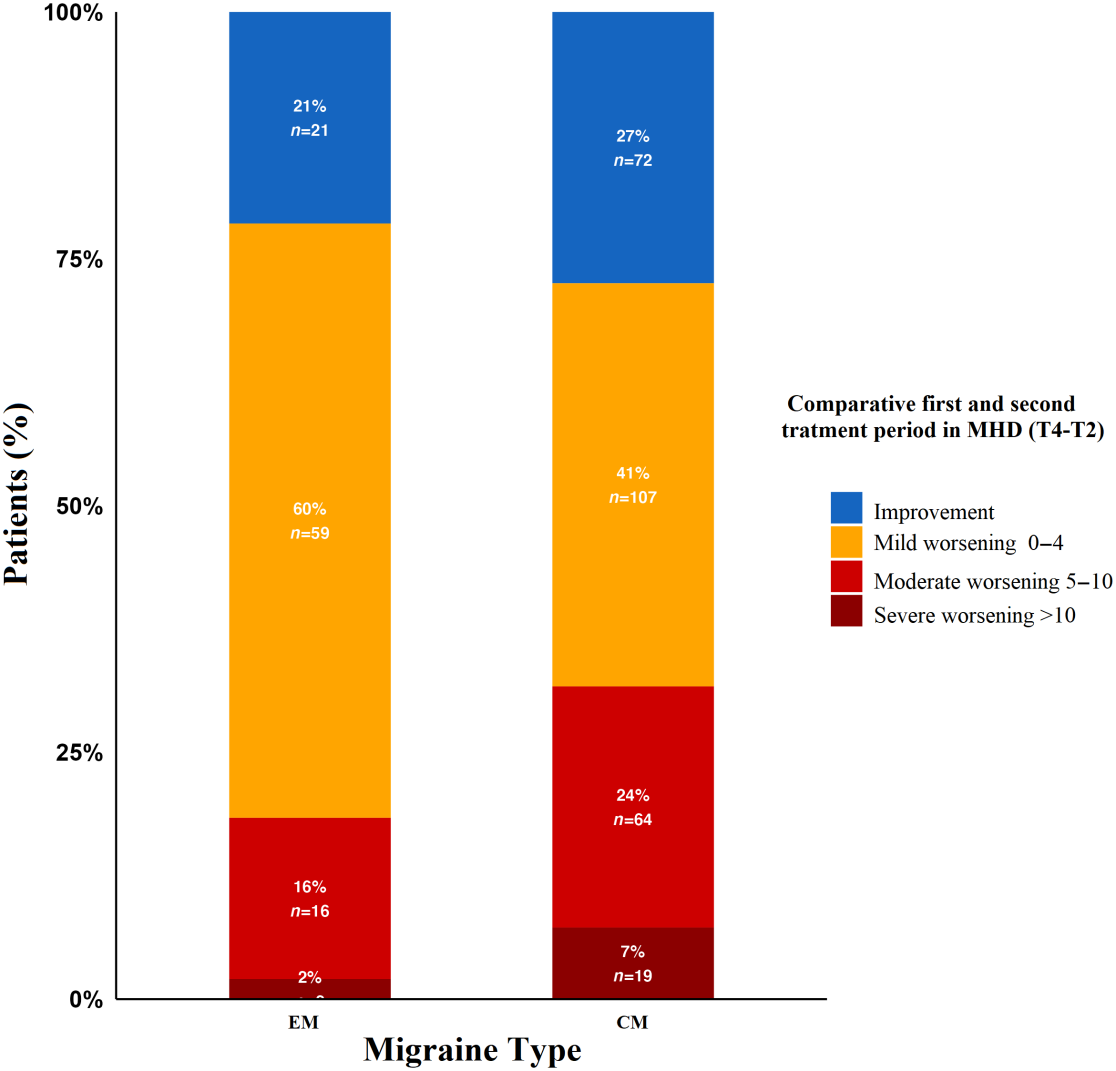
Bar graph of patients with high‐frequency episodic migraine (HFEM) and chronic migraine (CM) distributed in the classification to compare second period to first period treatment.

Afterwards we evaluated the second treatment period response in comparison with the first period. With regard to MHD, 93 patients (25.8%) showed the same improvement. Of the patients with worsening MHD, 166 (46%) had mild worsening, 80 (22%) moderate and 21 (5.8%) severe. In terms of MMD, 101 (29.2%) demonstrated an equivalent improvement, while 198 (57%) had a mild worsening, 38 (10.9%) had moderate worsening, and 10 (2.9%) had severe worsening considering the above‐mentioned ranges. These findings, in conjunction with their detailed breakdown by variables, are shown in Table 3 and ilustrated in Figure 3. Notably, patients who experienced migraine at a younger age exhibited worsening in MHD (*p* = 0.036) and MMD (*p* = 0.001) compared to the first period (Figure 4). Also, the group of patients with CM included a higher percentage of individuals classified as moderate to severe non‐responders in MHD (*p* = 0.007) and MMD (*p* = 0.026), as shown in Figure 5.

**TABLE 3:** Differences between outcomes in the two periods of treatment.

	Improvement (≤0)		Mild worsening (1–4 days)		Moderate worsening (5–10 days)		Severe worsening (>10 days)		*p* value	
	MHD	MMD	MHD	MMD	MHD	MMD	MHD	MMD	MHD	MMD
*N* total	93 (25.8%)	101 (29.1%)	166 (46%)	198 (57%)	80 (22%)	38 (10.9%)	21 (5.8%)	10 (2.9%)	>0.9	>0.9
Sex: female, *n* (%)	68 (76%)	76 (78%)	138 (83%)	164 (83%)	67 (84%)	30 (79%)	20 (95%)	10 (100%)	0.2	0.3
Age at migraine onset, years (IQR)	20 (14.6)	20.1 (14.9)	17.9 (9.5)	17.6 (8.7)	16.3 (13.6)	15.5 (14.6)	18.9 (18.5)	13.2 (8.5)	0.04	0.01
Age at anti‐CGRP MAb start, years (IQR)	48.7 (11.8)	48.7 (12.7)	48.8 (12.9)	48.5 (12.6)	48.3 (15.4)	47 (14.8)	52 (15.7)	55.2 (11.6)	>0.09	0.5
Migraine type: CM	72 (77%)	83 (82%)	107 (64%)	134 (68%)	64 (80%)	30 (79%)	19 (90%)	9 (90%)	0.007	0.026
NSAID overuse	40 (43%)	42 (42%)	49 (30%)	60 (30%)	30 (38%)	12 (32%)	5 (24%)	5 (50%)	0.1	0.2
Opioids overuse	2 (2.2%)	3 (3.0%)	1 (0.6%)	0 (0%)	0 (0%)	0 (0%)	1 (4.8%)	1 (10%)	0.2	0.012
Triptans overuse	42 (45%)	46 (46%)	66 (40%)	83 (42%)	32 (40%)	11 (29%)	6 (29%)	5 (50%)	0.5	0.3
Time between discontinuation and reintroduction	4.5 (3)	4.5 (2.8)	4.4 (3.7)	4.2 (3.7)	4.8 (3.6)	4.8 (3.7)	4.3 (2.1)	4.4 (6.5)	0.14	0.8

1 2

## DISCUSSION

In our study, we focused on the effectiveness of anti‐CGRP MAb in patients who had previously responded successfully according to the physician in charge to a first period of treatment but experienced subsequent relapse upon discontinuation and who later restarted the medication, in real‐life settings. Effectiveness was assessed in terms of MMD and MHD at 3 months after treatment reintroduction. To date, this is the largest sample size study investigating the reintroduction of anti‐CGRP MAb.

Analysis of the effectiveness of the second treatment period showed that, measured by 30% response rate, 80% of patients were responders with regard to MMD and 75% with regard to MHD. Thus, we suggest that anti‐CGRP MAb therapy was effective after reintroduction in a second period of treatment. In the study by Raffaelli et al. [16], 72.8% of patients had a 30% response rate 3 months after reintroduction, which is in line with our results. Iannone et al. [17] had previously described that patients in whom anti‐CGRP MAb treatment was reintroduced because of headache worsening after treatment disruption showed a reduction in MMD 1 month after anti‐CGRP MAb resumption in comparison to the third month after the pause, but did not achieve the same reduction in the MMD as that achieved at the end of the 12th month of treatment before the pause. In a cohort of 40 patients, Gantenbein et al. also reported that patients with anti‐CGRP MAb resumption after 3 months free of anti‐CGRP MAb showed an improvement in 1 month with respect to treatment interruption but these authors did not make a comparison with the first treatment period in their analysis [13]. In another publication with a sample size of 57, the restart of such treatment is discussed, and the secondary objective of this study was to examine what happened to patients who reintroduced treatment with erenumab after discontinuing it due to headache worsening. Ten patients reintroduced the treatment and during the first 4 weeks, 80% were ≥50% responders. Subsequently, from Week 5 to Week 8, the numbers of MMD and MHD were similar to those in the four last weeks of the first period of treatment [12]. However, comparison of the responses in the second with those in the first period of treatment revealed significant differences. In the second period, analysis of MMD at 3 months showed that one third of the patients achieved an equivalent response to that achieved in first period, although another 53% experienced mild worsening. Thus, up to 18% of patients did not reach the previous response within 3 months, with moderate to severe worsening. Similar trends were observed for MHD. Discontinuation of treatment could lead to a deterioration in the quality of life for these patients, suggesting that this should be considered before making a decision to discontinue.

Regarding patient profile, our results highlight that earlier onset of migraine, CM, and NSAIDs and triptans overuse may be associated with poorer response after treatment reintroduction when compared to the first treatment period. Raffaelli et al. [16] reported that eight patients with CM compared to three with HFEM did not respond to reintroduction in their sample.

Our multivariate model aimed to unravel the intricate interaction of demographic and migraine‐specific factors influencing treatment response. This approach suggests that patients experiencing earlier onset of migraine, those with a CM diagnosis, and those with medication abuse (NAIDs or triptans) are at greater risk of poorer response in the second treatment period. Reinforcing the importance of early intervention in patients, we found that individuals with chronic headaches exhibit a diminished response to medications. In addition, our findings highlight this trend during the post‐reintroduction period. Some studies have suggested that presence of HFEM is a predictor of response in the elderly [22]. Other studies suggest that pain characteristics such as unilaterality or triptan abuse are predictive factors, which is not in line with our results [23].

Most previous studies have evaluated response after treatment reintroduction using an independent measure. An advantage of our study is that we categorized the improvement or worsening of headaches according to varying degrees of response severity in comparison to the first treatment period response. This classification offers a comprehensive understanding of different levels of treatment efficacy, allowing nuanced assessment of clinical outcomes. We opted for this method of classifying patients because the timing of reintroduction is determined by clinical worsening, which does not correlate with the number of migraine days the patient experienced prior to anti‐CGRP MAb initiation. Furthermore, it allows for a more accurate intra‐patient comparison, as there could be patients who experience improvement with the treatment but still have days of pain, given that patients start from different baseline pain days.

Independently of these methodological differences, all previous studies, similarly to ours, highlighted the high probability of not achieving an equally satisfactory response to a second anti‐CGRP MAb treatment period in the first months of resumption.

A limitation of our study was the 3‐month follow‐up period, which may be considered relatively short. However, the 3‐month follow‐up period may be important because it conveys a significant delay for patients who were responding to with anti‐CGRP MAbs treatment until returning to their previous frequency of migraine attacks which may deteriorate their quality of life. It should also be noted that this was a multicentre study, which provided a larger sample size but also led to heterogeneity in the study cohort, with different clinical criteria used for discontinuation and reintroduction due to different treating physicians and regulations.

Overall, our study contributes to the evolving understanding of the dynamics of anti‐CGRP MAb treatment, emphasizing the need for ongoing research into the long‐term effects of such therapies and of treatment discontinuation and reinitiation. While our results suggest that the efficacy of anti‐CGRP MAb in a second treatment period is maintained with a response rate of >30%, only one third of patients achieved an equivalent response to that achieved in the first treatment period at 3 months after resumption. Longer follow‐up studies are needed to determine whether patients regain their previous favourable response and the time required to do so, or whether they do not regain it.

## CONFLICT OF INTEREST STATEMENT

Alicia Gonzalez‐Martinez has received speaker honoraria from TEVA and Altermedica. Sonia Quintas has received honoraria from Lilly, Novartis, Exeltis, UCB Pharma, Bial and Altermedica. David García‐Azorín reports payment or honoraria for lectures from Teva, Lilly, Allergan‐Abbvie, Novartis and Lundbeck, and has served on an advisory board for Allergan‐Abbvie. Ángel Luis Guerrero‐Peral has received honoraria from Lilly, TEVA, Lundbeck, Pfizer, Novartis, Allergan‐Abbvie and Exeltis, and research support from Allegan‐Abbvie, Lilly and TEVA. Sonia Santos‐Lassaosa has received honoraria from Lilly, TEVA, Lundbeck, Pfizer, Novartis, Allergan‐Abbvie and Exeltis, and research support from Allegan‐Abbvie, Lilly and TEVA. Mariano Huerta‐Villanueva has received honoraria for participating on advisory boards and for collaborations as consultant for scientific communications, speaker and research support, as well as funding for travel and congress attendance expenses from Abbie‐Allergan, Novartis, Lilly, Almirall, Chiesi, Esai, Exeltis, Kern Pharma, Menarini, TEVA and Zambon. His research group has received research grants from Abbie‐Allergan, and has received funding for clinical trials from Lilly, Novartis and TEVA. Sergio Campoy Díaz has received honoraria from TEVA, Lilly, Novartis, Roche, UCB, Bial, Chiesi, Allergan, Esai, Kern Pharma, Biogen Idec, Merck, Janssen, Neuraxpharm and/or Sanofi. Alberto Lozano Ros has received speaker honoraria from TEVA. Antonio Sánchez‐Soblechero has received speaker honoraria from TEVA. Fernando Velasco Juanes has received speaker honoraria from TEVA. Robert Belvís Nieto, has received payment honoraria for lectures from TEVA, Lilly, Allergan‐Abbvie, Lundbeck, Pfizer, Noema, Biogen, Exeltis, Chiesi, Abbott, Novartis, Almirall and Merck. Juan Carlos García‐Moncó has received speaker honoraria from Lilly and Teva, and has served on an advisory board for UCB Biopharma SRL. María Rocío Álvarez Escudero has received speaker honoraria from Lilly, Novartis and Abbvie‐Allergan, and has served on advisory boards for Lilly. Ana Beatriz Gago‐Veiga has received honoraria from Lilly, Novartis, TEVA, Abbvie‐Allergan, Exeltis and Chiesi. The rest of the authors report no conflict of interest regarding this manuscript.

## Data Availability

The data that support the findings of this study are available from the corresponding author upon reasonable request.

## Footnotes

1
*Note*: Similar improvement or worsening (mild, moderate, or severe) in MMD and MHD based on different variables. First period versus second period treatment. Data are *n* (%) or median (IQR).

2 Abbreviations: anti‐CGRP MAb, monoclonal antibodies against calcitonin gene‐related peptide; CM, chronic migraine; IQR, interquartile rangeMHD, monthly headache days; MMD, monthly migraine days; NSAIDs, nonsteroidal anti‐inflammatory drugs.

## References

[1] Kung D , Rodriguez G , Evans R . Chronic migraine: diagnosis and management. Neurol Clin. 2023;41:141‐159. doi:10.1016/j.ncl.2022.05.005 36400552

[2] GBD Collaborators . Global, regional, and national incidence, prevalence, and years lived with disability for 310 diseases and injuries, 1990–2015. Lancet. 2016;388:1545‐1602. 27733282 10.1016/S0140-6736(16)31678-6 PMC5055577

[3] Hovaguimian A , Roth J . Management of chronic migraine. BMJ. 2022;379:e067670. doi:10.1136/bmj-2021-067670 36216384

[4] Schiano di Cola F , Bolchini M , Ceccardi G , et al. An observational study on monoclonal antibodies against calcitonin‐gene‐related peptide and its receptor. Eur J Neurol. 2023;30:1764‐1773. doi:10.1111/ene.15761 36856538

[5] Serra López‐Matencio JM , Gago‐Veiga AB , Gómez M , et al. Treatment of migraine with monoclonal antibodies. Expert Opin Biol Ther. 2022;22:707‐716. doi:10.1080/14712598.2022.2072207 35502612

[6] Santos‐Lasaosa S , Belvís R , Cuadrado ML , et al. Calcitonin gene–related peptide in migraine: from pathophysiology to treatment. Neurologia. 2022;37:390‐402. doi:10.1016/j.nrl.2019.03.013 35672126

[7] Russo AF , Hay DL . CGRP physiology, pharmacology, and therapeutic targets: migraine and beyond. Physiol Rev. 2023;103:1565‐1644. doi:10.1152/physrev.00059.2021 36454715 PMC9988538

[8] Silvestro M , Orologio I , Siciliano M , et al. Emerging drugs for the preventive treatment of migraine: a review of CGRP monoclonal antibodies and gepants trials. Expert Opin Emerg Drugs. 2023;28:79‐96. doi:10.1080/14728214.2023.2207819 37185047

[9] Torres‐Ferrus M , Gallardo VJ , Alpuente A , Caronna E , Giné‐Ciprés E , Pozo‐Rosich P . Patterns of response to anti‐calcitonin gene‐related peptide monoclonal antibodies during first 6 months of treatment in resistant migraine patients: impact on outcome. Eur J Neurol. 2023;30:1937‐1944. doi:10.1111/ene.15816 37038303

[10] González GL , García NG , de Miguel CC , et al. Consensus about monoclonal antibodies in migraine of the Headache Study Group of the Madrid Association of Neurology. Kranion. 2023;18:5‐20. doi:10.24875/KRANION.M23000050

[11] Sacco S , Bendtsen L , Ashina M , et al. European headache federation guideline on the use of monoclonal antibodies acting on the calcitonin gene related peptide or its receptor for migraine prevention. J Headache Pain. 2019;20:6. doi:10.1186/s10194-018-0955-y 30651064 PMC6734227

[12] De Matteis E , Affaitati G , Frattale I , et al. Early outcomes of migraine after erenumab discontinuation: data from a real‐life setting. Neurol Sci. 2021;42:3297‐3303. doi:10.1007/s10072-020-05022-z 33389227

[13] Gantenbein AR , Agosti R , Gobbi C , et al. Impact on monthly migraine days of discontinuing anti‐CGRP antibodies after one year of treatment – a real‐life cohort study. Cephalalgia. 2021;41:1181‐1186. doi:10.1177/03331024211014616 34000847 PMC8504406

[14] Raffaelli B , Terhart M , Overeem LH , et al. Migraine evolution after the cessation of CGRP(‐receptor) antibody prophylaxis: a prospective, longitudinal cohort study. Cephalalgia. 2022;42:326‐334. doi:10.1177/03331024211046617 34579559 PMC8988461

[15] Sacco S , Amin FM , Ashina M , et al. European Headache Federation guideline on the use of monoclonal antibodies targeting the calcitonin gene related peptide pathway for migraine prevention – 2022 update. J Headache Pain. 2022;23:67. doi:10.1186/s10194-022-01431-x 35690723 PMC9188162

[16] Raffaelli B , Terhart M , Mecklenburg J , et al. Resumption of migraine preventive treatment with CGRP(‐receptor) antibodies after a 3‐month drug holiday: a real‐world experience. J Headache Pain. 2022;23:40. doi:10.1186/s10194-022-01417-9 35350990 PMC8966337

[17] Iannone LF , Fattori D , Benemei S , Chiarugi A , Geppetti P , De Cesaris F . Predictors of sustained response and effects of the discontinuation of anti‐calcitonin gene related peptide antibodies and reinitiation in resistant chronic migraine. Eur J Neurol. 2022;29:1505‐1513. doi:10.1111/ene.15260 35098620

[18] Olesen J . Headache classification Committee of the International Headache Society (IHS) the international classification of headache disorders, 3rd edition. Cephalalgia. 2018;38:1‐211. doi:10.1177/0333102417738202 29368949

[19] Al‐Hassany L , Lyons HS , Boucherie DM , et al. The sense of stopping migraine prophylaxis. J Headache Pain. 2023;24:9. doi:10.1186/s10194-023-01539-8 36792981 PMC9933401

[20] Belgorodski N , Greiner M , Tolksdorf K , Schueller K , Flor M , Göhring L . Fitting distributions to given data or known quantiles. Repository CRAN ‐ R Project 2017.

[21] Brooks ME , Kristensen K , van Benthem KJ , et al. glmmTMB balances speed and flexibility among packages for zero‐inflated generalized linear mixed modeling. R J. 2017;9:378‐400. doi:10.32614/rj-2017-066

[22] Gonzalez‐Martinez A , Sanz‐García A , García‐Azorín D , et al. Effectiveness, tolerability and response predictors of preventive anti‐CGRP mAbs for migraine in patients over 65 years old: a multicenter real‐world case‐control study. Pain Med. 2023;pnad141. doi:10.1093/pm/pnad141. Online ahead of print. 37847661

[23] Sánchez‐Rodríguez C , Gago‐Veiga AB , García‐Azorín D , Guerrero‐Peral ÁL , Gonzalez‐Martinez A . Potential predictors of response to CGRP monoclonal antibodies in chronic migraine: real‐world data. Curr Pain Headache Rep. 2023. doi:10.1007/s11916-023-01183-6. Online ahead of print. 37874459

